# A comparative study of ultrasound-guided puncture biopsy combined with histopathology and Xpert MTB/RIF in the diagnosis of lymph node tuberculosis

**DOI:** 10.3389/fpubh.2022.1022470

**Published:** 2023-01-10

**Authors:** Xiangyu Meng, Hongxiang Fu, Weina Jia, Ying Wang, Gaoyi Yang

**Affiliations:** ^1^Department of Ultrasonography, The Second Affiliated Hospital of Zhejiang Chinese Medical University, The Second Affiliated Hospital of Zhejiang Chinese Medical University, Xinhua Hospital of Zhejiang Province, Hangzhou, Zhejiang, China; ^2^Department of Radiology, Zhejiang Provincial People's Hospital, Hangzhou, Zhejiang, China; ^3^Department of Ultrasonography, School of Medicine, Hangzhou Normal University, Hangzhou, China; ^4^Department of Ultrasonography, Hangzhou Red Cross Hospital, Hangzhou, Zhejiang, China

**Keywords:** lymph node tuberculosis, needle biopsy, Xpert, diagnosis, interventional radiology, ultrasound-guided puncture

## Abstract

**Background:**

Cervical tuberculous lymphadenitis (CTBL) is a disease often ignored in clinical work, and pathology and Xpert MTB/RIF (Xpert) are the commonly used methods for tuberculosis diagnosis. This study aimed to compare ultrasound-guided puncture biopsy combined with histopathology and Xpert in the diagnosis of lymph node tuberculosis.

**Methods:**

A total of 217 patients highly suspected for CTBL were retrospectively enrolled. All patients underwent ultrasound-guided puncture sampling. All samples were subjected to pathological examination and Xpert test. The sensitivity and specificity of the two methods were compared for all samples. The kappa value was calculated to assess the consistency of the pathological examination and Xpert test using comprehensive diagnosis as the gold standard. Receiver operating characteristic curves of the pathological examination, Xpert test, and their combination were generated, and the areas under the curve (AUCs) were calculated to compare the diagnostic value of the three methods.

**Results:**

The sensitivity and specificity of the pathological diagnosis of CTBL were 70.1 and 100%, respectively. The sensitivity and specificity of Xpert for CTBL diagnosis were 82.5 and 97.5%, respectively. The results of the pathological examination and Xpert test showed poor consistency in the diagnosis of CTBL, with a kappa value of 0.388. The AUC of the pathological diagnosis of CTBL was 0.850 (95% CI: 0.796–0.895), whereas that of Xpert was 0.900 (95% CI: 0.852–0.936), and the difference was statistically significant (*P* = 0.0483). The AUC of pathological examination combined with Xpert for the diagnosis of CTBL was 0.956 (95% CI: 0.920–0.979), and the difference between pathological examination combined with Xpert for the diagnosis of CTBL was statistically significant compared with pathological examination and Xpert alone, respectively (both *P* < 0.001).

**Conclusion:**

The diagnostic efficiency of Xpert test is higher than that of pathological examination, but its sensitivity is still not ideal for clinical diagnosis. According to this study, the consistency of Xpert test and pathological diagnosis is poor, and the combination of Xpert test and pathological diagnosis can significantly increase the diagnostic efficiency.

## 1. Introduction

According to the World Health Organization's “Global Tuberculosis Report 2022” (1), tuberculosis (TB) is the second most lethal infectious disease after coronavirus disease 2019 (COVID-19). The COVID-19 pandemic has reversed years of advancement in the global fight against TB. In 2021, 10.6 million (range: 9.9–11 million) new cases of TB were reported worldwide, which is higher is than that reported in previous years. *Mycobacterium tuberculosis (M. tuberculosis)* infection in human hosts typically manifests in the lungs and is known as pulmonary tuberculosis (PTB); however, TB can also affect other parts of the body, such as lymph node and bone tuberculosis, this type of TB is known as extrapulmonary tuberculosis (EPTB). For a long time, with the global programmatic approach for TB control mainly focusing on PTB, EPTB remained a largely neglected disease. EPTB accounts for 15–20% of all cases of TB, and tuberculous lymphadenopathy is the most common form of EPTB, accounting for 35% of all cases of EPTB (2). Drug resistance to rifampicin in EPTB has been reported to be 4.8–21.2% (3, 4).

In our clinical study, we found that many patients with cervical tuberculous lymphadenitis presented to the hospital only due to a neck mass. Due to the lack of specific manifestations of systemic symptoms and small amount of bacteria in the lesions, the diagnosis of CTBL is often challenging, which hinders the clinical effectiveness of routine testing, and may ultimately lead to delayed diagnosis and poor prognosis. In particular, the formation of fistula in the late stage of the disease, which will cause great harm to the patient's physical and mental health.

There are several ways to diagnose TB, mainly relying on pathology, molecular biology, and bacteriology techniques. Each technique has its own advantages and disadvantages. Histopathology primarily makes diagnoses based on the presence of caseous necrosis, Langerhans cells, and tuberculous granuloma lesions; however, it can also misdiagnose non-tuberculous mycobacterium infection as tuberculosis infection.

Although culture testing remains the gold standard for TB diagnosis, the sensitivity of culture testing can vary from 30 to 80% in different extrapulmonary specimens, and it usually takes 2–8 weeks to receive the results, which is too slow for making treatment decisions (5). The sensitivity and specificity of Polymerase chain reaction (PCR) were 33–100% and 67–100%, respectively (6).

In 2013, the World Health Organization recommended the Xpert *Mycobacterium tuberculosis*/rifampicin resistance (MTB/RIF) assay (Cepheid, Sunnyvale, CA, USA) for the rapid diagnosis of extrapulmonary tuberculosis (7). This method can complete nucleic acid extraction, amplification, and fluorescence detection of *M. tuberculosis* in a proprietary reaction box and report the presence of *M. tuberculosis* complex infection and drug resistance to rifampicin within 100 min. It has been reported that the specificity of Xpert in the diagnosis of EPTB is 98–99.8%; however, its sensitivity (27–100%) varies significantly due to various sample sources and the reference gold standard (8, 9), making the quality of lymph node specimens particularly crucial to demonstrate. Contrast-enhanced ultrasound (CEUS) is sensitive to the display of lymph node microcirculation, which is conducive to high-quality sampling of needle biopsy, and improves diagnostic accuracy (10).

Therefore, in this study, we explore the value of ultrasound-guided needle biopsy combined with histopathology and Xpert detection in the diagnosis of lymph node tuberculosis to improve the time and efficiency of clinical diagnosis.

## 2. Materials and methods

### 2.1. Patients

This retrospective study was conducted in the Hangzhou Cross Hospital, Zhejiang Province, China. The 1975 Declaration of Helsinki's ethical principles were followed by the study protocol. This retrospective study received approval from the local ethics committee, and all patients provided their informed consent.

A retrospective analysis was performed on 217 patients who had high clinical suspicion of having CTBL from January 2019 to January 2021 (in this study, “patients with a high clinical suspicion of CTBL” were defined as patients who were punctured in a specific interventional ward). There were 92 males and 125 females, ranging in age from 18 to 82, with a median age of 34 (25, 52) years. Each patient's largest lymph node was selected as the study object. The inclusion criteria were as follows: the patients were (1) aged ≥18 years; (2) were at a high clinical suspicion of CTBL; (3) did not receive anti-tuberculosis treatment before the ultrasound-guided needle biopsy; (4) and be able to undergo both CEUS and ultrasound-guided needle biopsy. The exclusion criteria were as follows: (1) non-determination cases marked as “invalid,” “error,” or “no result” on Xpert software; (2) the lack of any definite diagnosis or follow-up to the final diagnosis.

### 2.2. Instruments and methods

#### 2.2.1. Instruments

The Philips iU22 color Doppler ultrasound diagnostic instrument, the L9-3 broadband linear array probe, frequency of 3–9 MHz. Ultrasound contrast agent (i.e., SonoVue lyophilized powder, Bracco Diagnostics Inc., Italy), 5 mL isotonic saline, 18 G needle biopsy needle (Bard, Covington, KY, USA), disposable 5 mL syringe (with needle 0.7 mm), disposable 10 mL syringe (with needle 1.2 mm), and BD needle.

#### 2.2.2. Methods

**Figure 1:**
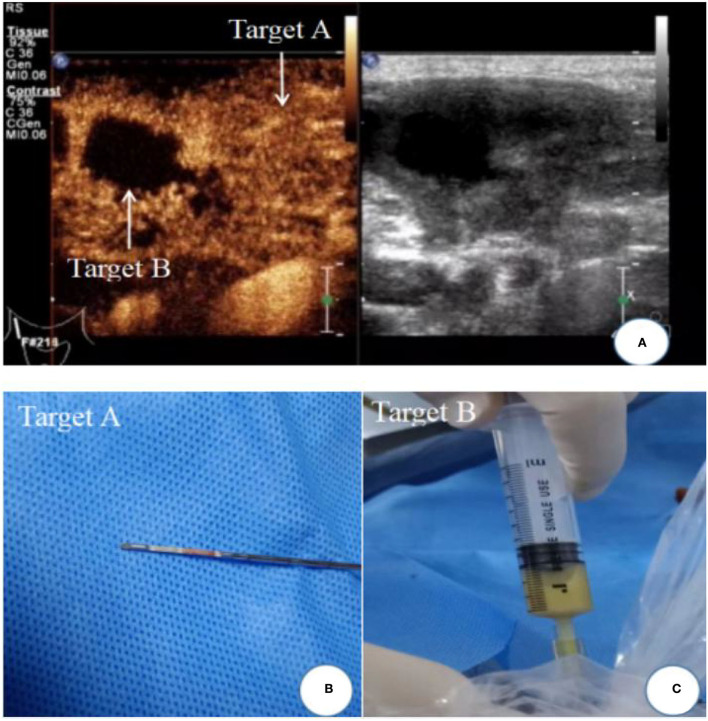
Ultrasound image of cervical lymph node tuberculosis. **(A)** Contrast-enhanced ultrasound showed uneven enhancement of lymph nodes, Target A: uneven enhancement areas; Target B: non-enhanced areas of caseous necrosis; **(B)** biopsy tissue specimen of enhanced area; **(C)** area without enhancement with fine-needle aspiration caseous necrosis.

The patient was instructed to remain supine and was examined on ultrasound by a board-certified radiologist after the machine settings had been optimized. Grayscale imaging, color Doppler imaging, and CEUS imaging were all used during the examination. Concerning the CEUS-guided needle biopsy, while an L9-3 linear array transducer (frequency range, 3.0–9.0 MHz) was used, a real-time contrast imaging software called pulse inversion harmonic imaging, supported by the Philips Healthcare System, was employed; this allowed contrast-enhanced imaging with low acoustic power [mechanical index (MI), 0.06]. The contrast agent SonoVue (59 mg) was mixed with 5 ml 0.9% normal saline. Target lymph nodes were first located through a conventional ultrasound scan of the lesion area. The data was then measured at the maximum cross-section of the target lesion. Following a licensed nurse's injection of the contrast agent (2.4 ml), CEUS images were shown in split-screen mode. The CEUS image display should last for at least 3 min. Each patient's images and videos were saved on a hard disk for further analysis. Following CEUS, the characteristics of CEUS images were used to decide where and how to puncture the biopsy. At the same time, samples from the same patient were collected. The local skin area was routinely disinfected, followed by local layer-by-layer anesthesia with 2% lidocaine hydrochloride, before the needle biopsy. The biopsy was carried out by the same board-certified radiologist with >10 years of experience in ultrasound-guided interventional procedures using a biopsy sampling device (Bard, Covington, KY, USA) in its 18G type (i.e., the diameter of the cutting needle was 1.2 mm; the length of sampling notch should be 1 or 2 cm based on the size of the lesion) within a real-time monitor through conventional ultrasound. Major blood vessels and tissues should be avoided during the needle biopsy. The enhanced region was subjected to a coarse-needle biopsy. From each case, two to four tissue samples were taken. Depending on the location and depth of the lesion, a 5- or 10-mL syringe with a matching needle or BD needle was used for fine-needle aspiration of the areas without enhancement. According to the volume of liquid in the lesion, aspirations of 1–10 mL of liquid were made (Figure 1).

### 2.3. Specimen fixation

#### 2.3.1. Specimens for pathological examination

To preserve the tissue strips collected from the needle trough, a 10% formalin-fixed solution was added. The 95% ethanol solution smear was used to fix the fine-needle aspirate.

#### 2.3.2. Sample submission for Xpert

The tissue strips that were taken out from the needle trough or the liquid pumped by fine-needle aspiration were placed into sterile specimen boxes and delivered to the tuberculosis laboratory for Xpert testing.

The pathological examination and Xpert test were carried out simultaneously once there was enough tissue collected. When there were not enough tissue samples (≤ 2 tissues), the samples were sent for pathological examination first, and the aspirate was sent to the laboratory for Xpert examination. In the event that tissue collection was unsuccessful, a portion of the aspirate was sent for testing to Xpert and a portion of the aspirate smear was sent for pathological examination.

### 2.4. Criteria for successful puncture sampling

The collection of at least 2–4 tissue strips (complete tissue), with each strip length > 5 mm (total length 10–20 mm or longer), is regarded as a successful core needle biopsy (11, 12).

### 2.5. Composite reference standard for CTBL

The WHO consolidated guidelines on tuberculosis (13) and the guidance on Tuberculosis Clinical Diagnostic Guidelines (14) of the UK National Agency for Health and Clinical Excellence served as the foundation for the Composite reference standard (CRS) for CTBL used in this study. The following were the CTBL criteria for comprehensive diagnosis: (1) cervical lymph node tuberculosis indicated by conforming clinical symptoms and imaging (including ultrasound, computed tomography, and magnetic resonance imaging); (2) pathological examination of the lesion specimen signifying cervical lymph node tuberculosis; (3) *M. tuberculosis* infection confirmed by *M. tuberculosis* culture, Xpert test, and other related diagnostic techniques; and (4) effective diagnostic treatment or regular anti-tuberculosis drug treatment for more than 6 months, with resolution or marked improvement of the corresponding clinical symptoms. Cervical lymph node tuberculosis is diagnosed if any two of the aforementioned conditions are true.

### 2.6. Pathological diagnostic criteria

#### 2.6.1. Category I diagnosis

A clear diagnosis of the disease, such as tuberculosis and lymphoma, can be directly made.

#### 2.6.2. Category II diagnosis

The diagnosis suggests the presence of a disease, such as chronic granulomatous inflammation, and the diagnosis usually takes the form of: tuberculosis considered, the likelihood of tuberculosis is high.

#### 2.6.3. Category III

Descriptive diagnosis of microscopic observations without diagnostic bias.

#### 2.6.4. Category IV diagnosis

No diagnosis can be made.

According to pathological diagnostic criteria (15), Categories I and II indicate pathologically confirmed tuberculosis, while Categories III and IV indicate no pathologic diagnosis of tuberculosis. Two hundred and seventeen patients were divided into pathologically confirmed and pathologically unconfirmed cases based on pathological diagnostic criteria.

### 2.7. Xpert diagnostic criteria

The Xpert MTB/RIF Operating Instructions Procedure was followed when conducting the Xpert test (16). The Instrumentation Testing Test Kit was provided by the China Global Fund Tuberculosis Project, and two experienced laboratory physicians using professional software to interpret the outcomes as follows: (1) negative; (2) positive and susceptible to rifampicin; and (3) positive and rifampicin-resistant.

### 2.8. Statistical analysis

All data were statistically analyzed using the SPSS Statistics software (SPSS version 22.0, IBM Corporation, Armonk, NY, USA) and MedCalc Statistical Software version 19.0.4 (MedCalc Software bvba, Ostend, Belgium). For continuous variable data, Shapiro–Wilk test was employed to determine normality, and the normally distributed continuous variables were expressed as mean ± standard deviation. Non-normally distributed continuous variables were expressed as median (interquartile range). The kappa value was used to express the consistency between pathological examination and the Xpert test. The receiver operating characteristic curves of pathological examination, Xpert test, and the combination of the two methods were plotted based on the gold standard of comprehensive diagnosis, and the areas under curve (AUCs) were calculated. The diagnostic values of the three methods were then compared.

## 3. Results

### 3.1. General results

A total of 217 lymph nodes were analyzed. The lengths of lymph nodes ranged from 1.2 cm to 5.3 cm, with an average of 2.95 ± 0.95 cm. A total of 177 CTBL cases and 40 cases of the non-tuberculous disease were diagnosed by CRS. One hundred and twenty-four CBTL cases and 93 of non-tuberculous disease were made as a result of the pathological examination. One hundred and forty-seven cases of CTBL and 70 cases of the non-tuberculous disease were identified by Xpert. Additionally, Table 1 includes information on the diagnostic outcomes of the other components of comprehensive diagnostics.

**Table 1:** Number of cases of lymph node tuberculosis diagnosed by different methods.

**Methods**	**Cultures**		**Pathological examination**		**Xpert**			**PCR**		**Empirical treatment and follow-up**		**Clinical treatment and imaging**	
**Results**	+	−	+	−	+		−	+	−	+	−	+	−
					**RIF susceptible**	**RIF susceptible**							
The number of cases	56	121	124	53	138	9	30	110	67	9	2	109	68

One hundred and seventy-seven cases diagnosed with CTBL based on CRS. “+” means confirmed lymph node tuberculosis. “−” indicates that lymph node tuberculosis has not been diagnosed.

RIF, Rifampicin; PCR, Polymerase Chain Reaction.

### 3.2. Results of ultrasound-guided puncture sampling

The success rate of ultrasound-guided tissue sampling was 94.5% (205/217).

### 3.3. Positive rate of pathological examination, Xpert test, and their combined diagnosis

The positive rate of pathological diagnosis was 70.1% (124/177), that of Xpert diagnosis was 82.5% (146/177), and that of the combination of pathological examination and Xpert test was 93.8% (166/177).

### 3.4. Comparison of sensitivity and specificity of pathological examination, Xpert test, and their combined diagnosis

The sensitivity and specificity of the pathological diagnosis of CTBL were 70.1 and 100%, respectively, those of Xpert were 82.5 and 97.5%, respectively, and those of the combination of pathological examination and Xpert test were 93.8 and 97.5%, respectively (Table 2).

**Table 2:** Comparative results of pathological examination, Xpert test, and their combination in terms of sensitivity and specificity (%).

**Methods**	**Sensitivity**	**Specificity**
Pathology	70.1	100
Xpert	82.5	97.5
Combination	93.8	97.5
χ^2^	30.947	–
*P*	< 0.001	1.000

Fisher's exact test was used to compare the specificity of pathological examination, Xpert test, and their combined diagnosis. The sensitivity of pathological examination, Xpert test and their combination was compared, and the differences between them was statistically significant (pathology vs. Xpert: χ^2^ = 7.554, *P* < 0.006; pathology vs. combination: χ^2^ = 33.645, *P* < 0.001; Xpert vs. combination: χ^2^ = 10.806, *P* = 0.001).

### 3.5. Pathological examination and Xpert test diagnosis CTBL consistency comparison

The CTBL found by pathological examination and Xpert had a kappa value of 0.388 using comprehensive diagnosis as the gold standard. A kappa value < 0.4 indicates poor consistency between them.

### 3.6. Comparison of pathological examination, Xpert test, and their combined diagnostic efficacy

**Figure 2:**
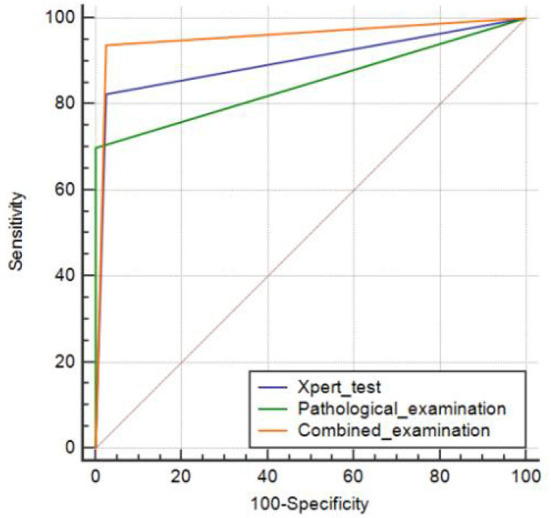
Areas under the receiver operating characteristic curve plotted to determine the diagnostic values of pathological examination alone, Xpert examination alone, and the combination of the two.

Using a comprehensive diagnosis as the gold standard, the AUC of pathological examination for CTBL was 0.850 (95% CI: 0.796–0.895), whereas that of Xpert was 0.900 (95% CI: 0.852–0.936), and the difference was statistically significant (*P* = 0.0483). The AUC of the combined diagnosis of CTBL by pathology and Xpert was 0.956 (95% CI: 0.920–0.979), and the difference was statistically significant (both *P* < 0.001) when compared with each pathological examination alone and Xpert alone (Figure 2).

## 4. Discussion

To compare the differences between the two methods for diagnosing lymph node tuberculosis, samples were obtained using an ultrasound-guided technique and pathological examination and the Xpert test were run concurrently. In the diagnosis of lymph node tuberculosis, the results demonstrated that the diagnostic value of Xpert was superior to that of pathological examination, but the consistency of the two methods was subpar. When the two methods were combined instead of just the examination alone, it was discovered that the diagnostic effectiveness could be could be significantly improved.

The most commonly affected sites of extrapulmonary tuberculosis involvement are lymph nodes (17), and surgical resection pathology has always been the gold standard for the diagnosis of lymph node lesions (11, 18). However, due to excessive trauma or inadequate wound healing, surgical resection biopsy is usually not widely accepted, and some lymph nodes with deep locations or locations adjacent to important organs are challenging to operate on (19). Contrarily, CEUS-guided needle biopsy is well-accepted by patients, safe, and well-known in a variety of fields (20, 21).

In order to clearly distinguish between active tissue and necrotic area and precisely locate the target, CEUS is highly sensitive and can visualize the microscopic structure and low-speed microcirculation blood perfusion in tissues (22, 23). The difficulty of pathological diagnosis brought on by insufficient sampling can be lessened by sampling lymph node perfusion areas with a contrast agent because it has good tissue integrity (24, 25). The success rate of biopsy sampling after CEUS in the current study was up to 94.5% (205/217), which satisfied the requirements of pathological diagnosis for tissues. The remaining 12 cases with failed sampling, were detected using CEUS, which revealed either almost no contrast agent perfusion or sparse small dots and patches of contrast agent perfusion throughout the entire lymph node. In the unenhanced area, fine-needle aspiration was used to collect samples ranging in size from 1 to 10 mL. Although it is not appropriate for pathological examination, this area has some benefits for Xpert. Regardless of the nature and texture of the sample, the area can be aspirated with a fine needle and the sample sent for an Xpert test.

Pathology and Xpert both examined each lymph node sample that was obtained with the help of ultrasound guidance. One of the current molecular biological detection techniques for tuberculosis diagnosis is Xpert. It is a fully automatic nucleic acid amplification detection technology based on semi-nested real-time polymerase chain reaction technology. This method primarily creates primers and probes for the 81-bp rifampin-resistance-determining region interval of rpoB of *M. tuberculosis* and then tests whether the test sequence has mutations. If a patient has both TB infection and rifampicin resistance, Xpert then indicates this (26). Since *M. tuberculosis* is present in the sample, regardless of whether the specimen is alive or not, the Xpert diagnostic method tests the gene sequence. Xpert has high sensitivity as a result. By successfully detecting the target specimen in tissue strips and aspirate, Xpert is not constrained by the characteristics of specimens, demonstrating its broad clinical applicability. However, 30 cases of CTBL in the current study were not successfully diagnosed. Different sampling areas may have resulted in different test results, given insufficient tissue or fluid samples extracted by ultrasound-guided coarse-needle biopsy or fine-needle aspiration and uneven distribution of bacilli in the lesions. On the other hand, pathological diagnosis of CTBL also has some limitations. The previous “gold standard” for the definition of lymph node tuberculosis was: granuloma formation, caseous necrosis, and the presence of Langerhans giant cells. A series of “classic” histopathological features of TB cannot effectively distinguish between *M. tuberculosis* and other mycobacteria. Furthermore, central caseous necrosis in small biopsy samples are not always obvious, and caseous necrosis may also exist in other granulomatous lesions (27). This could also be the cause of some pathological findings in the current study that can only present a descriptive diagnosis and cannot be confirmed, resulting in lower diagnostic efficiency than Xpert.

When diagnosing CTBL, Xpert had a sensitivity rate of 82.5%, compared to a 70.1% rate for pathological analysis. The diagnostic efficacy of the two by itself was not optimal for clinical diagnosis, even though Xpert was more helpful in the diagnosis of CTBL. The diagnostic effectiveness was significantly increased by the combined diagnosis of CTBL, and the difference was statistically significant. We advise that samples that are highly suspected of having lymph node tuberculosis be sent to hospitals with relevant conditions or designated tuberculosis diagnostic and treatment centers, for both a pathological examination and an Xpert test at the same time. The accuracy of the CTBL diagnosis will be significantly increased by combining the two diagnostic methods.

This study has certain limitations. First, the needle biopsy heavily depends on the operator's experience and technique, and if different operators will have certain influence on the results were not evaluated or accounted for. Second, the Xpert test was utilized in this study due to equipment restrictions. Xpert Ultra is an upgraded version of Xpert that has two different multi-copy amplification targets and may offer increased sensitivity due to its lower bacterial detection floor. Third, there are not enough cases in this study, which would have left it vulnerable to sampling error.

## 5. Conclusion

The findings of this study demonstrate that CEUS-guided puncture biopsy of lymph node tuberculosis has a high sampling success rate, which is helpful to lessen the diagnostic challenges brought on by the quality of sampling. Although the Xpert test has a higher diagnostic effectiveness than a pathological examination, its sensitivity is still not ideal for clinical diagnosis. According to this study, the consistency of Xpert test and pathological diagnosis is poor, and the combination of Xpert test and pathological diagnosis can significantly increase the diagnostic efficiency.

## Data availability statement

The original contributions presented in the study are included in the article/supplementary material, further inquiries can be directed to the corresponding author.

## Ethics statement

The studies involving human participants were reviewed and approved by the Ethical Committee of Hangzhou Red Cross Hospital. The patients/participants provided their written informed consent to participate in this study.

## Author contributions

XM and HF performed study design, information collection, statistical analysis, and manuscript editing. GY guided study design, reviewed images, and revised manuscript. WJ collected images and clinical information. YW made contributions to the data re-verification, sorting, and statistics. All authors contributed to the article and approved the submitted version.
